# Improvements in upper extremity isometric muscle strength, dexterity, and self-care independence during the sub-acute phase of stroke recovery: an observational study on the effects of intensive comprehensive rehabilitation

**DOI:** 10.3389/fneur.2024.1442120

**Published:** 2024-10-23

**Authors:** Řasová K., Martinková P., Vařejková M., Miznerova B., Hlinovská J., Hlinovský D., Iskendri D., Lebdušková L., Vojíková R., Zakouřilová J., Běhounek J., Musil V., Philipp T.

**Affiliations:** ^1^Department of Rehabilitation Medicine, Third Faculty of Medicine, Charles University, Prague, Czechia; ^2^Department of Statistical Modelling, Institute of Computer Science of the Czech Academy of Sciences, Prague, Czechia; ^3^Department of Rheumatology and Physiotherapy, Third Faculty of Medicine, Charles University and Thomayer University Hospital, Prague, Czech Republic; ^4^Department of Rehabilitation and Sports Medicine, Second Medical Faculty, Charles University and University Hospital Motol, Prague, Czech Republic; ^5^Department of Neurology, Third Faculty of Medicine, Charles University and Thomayer University Hospital, Prague, Czech Republic; ^6^Center of Scientific Information, Third Faculty of Medicine, Charles University, Prague, Czechia

**Keywords:** ischemic stroke, rehabilitation, physiotherapy, isometric grip strength, maximum strength during key, tripod, and tip-tip pinch, dexterity

## Abstract

**Background:**

Stroke often impairs upper extremity motor function, with recovery in the sub-acute phase being crucial for regaining independence. This study examines changes in isometric muscle strength, dexterity, and self-care independence during this period, and evaluates the effects of a comprehensive intensive rehabilitation (COMIRESTROKE).

**Methods:**

Individuals in sub-acute stroke recovery and age- and sex-matched controls were assessed for pre- and post-rehabilitation differences in primary outcomes (grip/pinch strength, Nine Hole Peg Test [NHPT], Action Research Arm Test [ARAT]). COMIRESTROKE’s effects on primary and secondary outcomes (National Institute of Health Stroke Scale [NIHSS], Modified Rankin Scale [MRS], Functional Independence Measure [FIM]) were evaluated. Outcomes were analyzed for dominant and non-dominant limbs, both regardless of impairment and with a focus on impaired limbs.

**Results:**

Fifty-two individuals with stroke (NIHSS 7.51 ± 5.71, age 70.25 ± 12.66 years, 21.36 ± 12.06 days post-stroke) and forty-six controls participated. At baseline, individuals with stroke showed significantly lower strength (dominant grip, key pinch, tip-tip pinch, *p*_adj_ < 0.05), higher NHPT scores (*p*_adj_ < 0.05), and lower ARAT scores (*p*_adj_ < 0.001). COMIRESTROKE led to improvements in dominant key pinch, non-dominant tip-tip pinch, NHPT, and both dominant and non-dominant ARAT (*p*_adj_ < 0.05). Notably, non-dominant key pinch improved significantly when considering only impaired hands. Pre- and post-test differences between groups were significant only for ARAT (both limbs), even after adjustment (*p*_adj_ < 0.05). All secondary outcomes (NIHSS, MRS, FIM) showed significant improvement post-COMIRESTROKE (*p*_adj_ < 0.001).

**Conclusion:**

Individuals with stroke exhibit reduced muscle strength and dexterity, impairing independence. However, comprehensive intensive rehabilitation significantly improves these functions. Data are available from the corresponding author upon request and are part of a sub-study of NCT05323916.

## Introduction

Stroke is a leading cause of physical impairments (1, 2). Up to 80% of post-stroke survivors have impairment of the upper extremity (2–6). Its most common symptom is paresis (3, 7, 8), which is characterized by reduced muscle strength (caused by reduced motor unit recruitment and muscle changes as atrophy (8)) and subsequently by loss or limitation of function in muscle control or movement or mobility (3, 5, 8–12) which can subsequently negatively affect self-sufficiency and quality of life (2, 8, 13). Due to upper extremity impairment, more than 50% of individuals post-stroke require assistance (usually mild to moderate) in dressing or bathing, and the majority require full assistance in some activities of daily living, such as meal preparation or housekeeping. Only a few of them can return to work and devote themselves to family and leisure activities as they did before the stroke (1, 7, 14, 15). The therapeutic influence of upper limb impairment is, therefore, very important from the point of view of maintaining functional independence (2, 5).

Promoting functional recovery of the impaired upper extremities is one of the major goals of stroke rehabilitation (3). Most motor recovery occurs early, typically plateauing around 3 months after stroke (16–18), due to processes of motor control and learning that are shared with the mechanisms of adaptive functional reorganization during spontaneous recovery (3, 19–21). These recovery processes can be enhanced through appropriate rehabilitation, which involves applying various intrinsic or extrinsic stimuli (6) aimed at remodeling the brain’s plastic and adaptive processes (22). Appropriate rehabilitation encompasses a range of techniques specifically designed to improve upper limb function following a stroke (23, 24). Such approaches include bilateral training (4), constraint-induced movement therapy (25), electrical stimulation (26), repetitive task training (15, 23), reaching distance and speed instructions (27), and robotics (1).

While key rehabilitation principles, such as therapy intensity (23, 28–30) and comprehensiveness (24, 31, 32), are recognized as important, their effects on upper extremity function remain insufficiently explored, as most studies focus on therapies specifically targeting upper extremity function (1, 4, 15, 23). Therefore, this study aims to evaluate the impact of a comprehensive inpatient rehabilitation program (COMIRESTROKE) (33) among individuals in sub-acute stroke recovery.

We measured strength as a predictor of motor performance, functional ability, and recovery following rehabilitation. The methodology for assessing muscle strength in individuals with central motor neuron impairment is not straightforward. While some researchers consider isokinetic dynamometry to be the “gold standard” (34) due to its ability to capture the dynamic nature of functional tasks (7, 35), others advocate for evaluating isometric muscle strength as a more suitable approach (10, 16, 36). Isometric strength testing is often regarded as a more sensitive measure of motor performance, despite its inherent variability (7), because it is specifically designed to evaluate initial upper limb recovery and serves as a reliable prognostic indicator for future recovery (16). Additionally, it has demonstrated strong reliability and a close association with motor performance (10). Therefore, we opted for isometric muscle strength testing in our study to leverage these advantages.

On the other hand, hand-held dynamometry assesses only a single aspect of function; it can monitor the level of motor dysfunction and provide key information about the function of the motor cortex. However, the results cannot be generalized to functional movement ability or the capacity for rehabilitation (12). To provide a more comprehensive evaluation, we also included more functionally relevant tests (7) focused on dexterity, such as the Nine Hole Peg Test (NHPT) and the Action Research Arm Test (ARAT), in our primary outcomes.

Additionally, we were interested in exploring whether improvements in upper limb impairment would impact the ability to maintain independence. To address this, we included the Functional Independence Measure (FIM) as a secondary outcome.

Although the literature clearly indicates that upper extremity function and subsequent self-care independence are impaired in individuals with stroke, no study has yet directly compared clinical parameters with a control group. Therefore, in our study, we included individuals of similar age and sex without significant neurological or orthopaedic conditions as a control group. This approach aimed to strengthen the validity of our results and support the reliability of the measurements.

The goals of this study were:

1 To characterize the clinical involvement of the upper extremity in individuals during the sub-acute phase of stroke recovery and compare it with control groups.

We hypothesized that there would be significant differences in primary outcomes between individuals with stroke and the control groups. Additionally, we anticipated a correlation between primary and secondary outcomes in individuals with stroke.

2 To evaluate the effect of the COMIRESTROKE rehabilitation program.

We hypothesized that there would be no significant changes between the first and second measurements in the control group. In contrast, we expected to see significant improvements in primary outcomes before and after rehabilitation in individuals with stroke, with notable differences between the stroke and control groups.

## Methods and analysis

### Study design

This article analyses data collected between June 1, 2020 to July 31, 2023 as a sub-study of the NCT05323916 (33). Primary outcomes (isometric grip and pinch force, NHPT, and ARAT) were assessed twice: in individuals with stroke before and after 3 weeks of COMIRESTROKE, and in controls in the same interval without rehabilitation. Secondary outcomes (National Institute of Health Stroke Scale, the Modified Rankin Scale, and FIM) were investigated only in individuals with stroke before and after rehabilitation.

### Participants

Individuals in the sub-acute phase of stroke recovery hospitalised in the Stroke Center of the Thomayer University Hospital were included by a neurologist based on these *criteria*: adults (18–89 years) after the first ischemic stroke in the early sub-acute phase (16, 17), with a slight to moderately severe disability (2–5 on the Modified Rankin Scale). People with low levels of consciousness, severe cognitive decline, and severe medical problems with a poor prognosis (37) were excluded.

As controls, individuals hospitalized at the same hospital were selected by a medical doctor to be as similar as possible to the post-stroke individuals in terms of sex and approximate age. These controls were admitted for planned examinations or scheduled rehabilitation aimed at improving musculoskeletal pain due to vertebropathy or enhancing their physical or mental condition. Importantly, they were not hospitalized for any acute reason, and any serious neurological or orthopaedic diseases.

### Interventions

Individuals who experienced a stroke participated in the COMprehensive Intensive REhabilitation program after STROKE (COMIRESTROKE). This program, overseen by a medical doctor, provided personalized therapies tailored to each patient. The rehabilitation combined physiotherapeutic techniques (with at least 1 hour daily), occupational therapy, psychotherapeutic approaches, and logopaedic techniques, all delivered in an intensive format. The therapy sessions lasted 4 h each day, 6 days a week, over a 3-week period (38). The treatment in each session was led by educated and experienced therapists at the Department of Rheumatology and Rehabilitation, Third Faculty of Medicine, Charles University, and Thomayer University Hospital.

Controls underwent standard inpatient hospitalization without any pre-defined rehabilitation.

### Assessment

*Demographic and anamnestic data*, including sex, age, weight and height, handedness (39), and number of days after the stroke event, were obtained after enrolment in the study.

An independent and certified Clinical Evaluator assessed the primary and secondary outcomes before and after the COMIRESTROKE program.

#### Primary outcomes

Isometric grip strength was measured using a Jamar Hydraulic Hand Dynamometer (40). The measurement was conducted while the subject was seated on a chair with their lower limbs resting on the floor. The upper limbs were positioned with the arm in adduction, the elbow flexed at 90°, the forearm and wrist in a neutral position, and the fingers slightly extended. The dynamometer handle can be adjusted to five grip positions (9, 12, 14.5, 17, and 20 cm) to accommodate different hand sizes (41). In each of the five positions, the maximum isometric force was measured three times, and the average of these measurements was calculated. The highest value obtained across the positions was selected, with a higher value indicating better grip strength.

*Isometric maximum strength during key, tripod, and tip-tip pinch* were measured by a Pinch Gauge dynamometer. The measurement took place in the same position as during the measurement of isometric grip strength, only the examiner held the dynamometer to prevent it from falling. The isometric force was measured three times for each of three grip pinchs, and the average isometric force was recorded (a higher value means a better result) (40).

*Action Research Arm Test* is a 19-item observational measure assessing upper extremity performance (coordination, dexterity, and functioning). A higher score means better functioning (42).

*The Nine Hole Peg Test* is used to measure finger dexterity. A client takes the pegs from a container, one by one, and places them into the holes on the board as quickly as possible. Shorter times reflect better functioning (43).

#### Secondary outcomes

*The National Institute of Health Stroke Scale* measures stroke-related neurological deficit (44) on a 15-item scale from 0 to 42 (higher scores indicating greater severity).

*The Modified Rankin Scale* (45) is used to categorize the level of functional independence with reference to pre-stroke activities on a scale from 0 to 5 (higher scores indicating greater disability).

*Functional Independence Measure* (46) evaluates independence for self-care (e.g., sphincter control, transfers, locomotion, communication, social cognition). The higher the score (between 18 and 126), the more independent the person is in performing the task.

### Statistical analysis

We tested for differences in basic descriptive characteristics between individuals with stroke and controls. In the case of binary variables – sex and laterality, Pearson’s chi-squared test was used, while for continuous variables, two-sample t-tests were employed. Outcomes were presented separately for the dominant and non-dominant limbs. Differences between individuals with stroke and controls were first analyzed regardless of limb impairment, then with a focus on impaired limbs. A side was considered impaired if the neurologist assigned a minimum score of 1 on the Motor Function Arm scale, a sub-scale of the NIHSS, which ranges from 0 to 9.

Differences between individuals with stroke and controls in measured outcomes at baseline (after the first measurement) were tested using two-sample t-tests. All *p*-values were adjusted for multiple comparisons using the Benjamini-Hochberg correction.

The difference between pre-scores (first measurement) and post-scores (second measurement) was tested separately for both groups (individuals with stroke and controls) using paired t-tests. Then, two-sample t-tests were used to assess pre-post differences in scores between controls and individuals with stroke. Adjustment for multiple comparisons was applied.

To evaluate associations between measured outcomes at baseline for individuals with stroke, Pearson’s correlation coefficient *r* was used. The differences were evaluated as statistically significant if *p*_adj_ < 0.05. The analysis was performed using the free statistical software R, version 4.3.2 (37), and its corresponding packages.

## Results

### Participants’ characteristic

Table 1 shows the CONSORT diagram of participant recruitment. Fifty-two individuals with stroke (NIHSS 7.51 ± 5.71, age 70.25 ± 12.66 years, days after stroke onset 21.36 ± 12.06) and forty-six controls were included in the study. There were no significant differences between groups in basic characteristics (see Table 2). Fifteen individuals with stroke had impaired right side, 21 had left side, 4 had bilateral impairment, and 8 did not have detectable impairment on NIHSS. In four cases, information about impairment was not available. Among the 52 subjects, 18 had impairment in their dominant hand, 31 had no impairment, and 3 had unknown impairment status of their dominant hand. For the non-dominant hand, 28 subjects had impairment, 21 had no impairment, and 3 had unknown impairment status.

**Table 1:** The CONSORT diagram of participant recruitment.

Individuals with stroke		Controls
140	Screened	125
57	Met inclusive criteria	49
57	Consented to participate and underwent first examination	47
52	Completed the study	46
4 Discharged at one’s own request (subjective opinion that one does not need to be hospitalised/rehabilitated) 1 Parched rehabilitation due to Covid 19	Reasons for not completing the second measurement	1 Discharged before a second examination was organised

**Table 2:** Sample characteristics.

	Individuals with stroke		Controls		*p*-value*
	M	F	M	F	
Sex	28	24	21	25	0.544
	R	L	R	L	
Laterality	47	3	41	5	0.622
	R	L			
Affected side	20	27			
	Mean	SD	Mean	SD	
Age (years)	70.25	12.66	72.76	15.20	0.380
Weight (kg)	80.59	23.22	75.72	20.63	0.277
Height (cm)	168.63	13.97	169.28	17.86	0.842
Days after stroke	21.36	12.06			

*Testing difference between individuals with stroke and controls (in case of binary variables—sex and laterality, Pearson chi-squared test is used, for continuous variables two-sample *t*-test is used).

R, right; L, left; M, male; F, female; SD, standard deviation.

Among the subjects from experimental group, 47 were right-handed and 3 were left-handed before their stroke; hand dominance was not determined for 2 subjects. In the control group, 41 were right-handed and 5 were left-handed.

The clinical involvement of the upper extremity of individuals in the sub-acute phase of stroke recovery.

Individuals with stroke had more clinically affected both dominant (D) and non-dominant (ND) upper limbs, documented by significantly lower strength (grip D mean difference −4.99 ± 10.84, key pinch D mean difference −1.01 ± 1.97, tip-tip pinch D mean difference −0.96 ± 1.57, and tip-tip pinch ND mean difference −0.74 ± 1.77), higher value in NHPT D (mean difference 19.51 ± 26.45) and NHPT ND (mean difference 12.47 ± 17.04) and lower scores in ARAT D (mean difference −11.30 ± 15.09) and ARAT ND (mean difference −17.04 ± 18.72), see Table 3.

**Table 3:** Difference between individuals with stroke and controls in pre-test.

	Individuals with stroke			Controls			Individuals with stroke vs. controls			
	N	Mean	SD	N	Mean	SD	Diff	SD_both_	Stat	*p*-value_adj_
NHPT D	42	45.88	35.07	46	26.37	6.78	19.51	26.45	3.55	0.004
NHPT ND	32	40.70	22.94	46	28.23	8.32	12.47	17.04	2.94	0.013
grip D	45	20.55	9.51	46	25.54	11.57	−4.99	10.84	−2.25	0.046
grip ND	38	18.86	10.50	46	24.05	12.07	−5.19	11.61	−2.11	0.057
key pinch D	45	4.24	2.01	46	5.25	1.81	−1.01	1.97	−2.51	0.028
key pinch ND	38	4.49	2.73	46	4.80	1.99	−0.31	2.34	−0.58	0.612
tripod pinch D	44	2.84	1.50	46	3.15	1.29	−0.30	1.40	−1.02	0.371
tripod pinch ND	37	2.93	1.91	46	2.98	1.34	−0.05	1.61	−0.15	0.885
tip-tip pinch D	44	3.25	1.57	46	4.21	1.43	−0.96	1.57	−3.01	0.010
tip-tip pinch ND	35	3.10	1.89	46	3.84	1.62	−0.74	1.77	−1.85	0.091
ARAT D	50	45.70	19.48	46	57.00	0.00	−11.30	15.09	−4.10	0.001
ARAT ND	47	39.96	23.55	46	57.00	0.00	−17.04	18.72	−4.96	< 0.001

SD, standard deviation; adj, adjusted; D, dominant; ND, non-dominant; NHPT, Nine Hole Peg Test; ARAT, Action Research Arm Test.

Non-dominant NHPT (r = −0.45) non-dominant grip (r = 0.37), both key (r = 0.33 for dominant, r = 0.37 for non-dominant) and tip-tip pinch (r = 0.33 for dominant, r = 0.35 for non-dominant) correlated well with FIM.

### The effect of the COMIRESTROKE

COMIRESTROKE had a positive effect on primary outcomes: The improvement was significant in key pitch D (0.40 ± 0.94, *p*_adj_ = 0.027) and tip-tip pinch ND (0.39 ± 0.67, *p*_adj_ = 0.015), also, the mean NHPT ND decreased by 8.43 ± 18.46 (*p*_adj_ = 0.049), mean ARAT D increased by 3.36 ± 7.37 (*p*_adj_ = 0.015) and mean ARAT ND increased by 3.17 ± 6.39 (*p*_adj_ = 0.0015); also, secondary outcomes, NIHSS, MRS, and FIM improved significantly (*p*_adj_ < 0.001), see Table 4.

**Table 4:** Difference between individuals with stroke and controls in pre-test post-test differences.

	Individuals with stroke								Controls								Individuals with stroke vs. controls			
		PRE		POST		PRE-POST				PRE		POST		PRE-POST						
	N	Mean	SD	Mean	SD	Mean	SD	*p-*value_adj_	N	Mean	SD	Mean	SD	Mean	SD	*p-*value_adj_	Diff	SD_both_	Stat	*p-*value_adj_
NHPT D	38	46.77	36.79	36.46	13.82	−10.31	34.17	0.122	45	26.41	6.85	26.87	8.33	0.47	3.75	0.738	−10.78	23.74	−1.93	0.145
NHPT ND	29	41.97	23.76	33.55	10.66	−8.43	18.46	0.049	45	28.27	8.41	28.29	7.68	0.02	5.24	0.976	−8.45	12.83	−2.40	0.090
grip D	41	20.70	9.50	21.57	10.09	0.88	2.93	0.122	45	25.66	11.67	25.41	12.68	−0.25	4.68	0.899	1.13	3.96	1.36	0.307
grip ND	35	18.33	10.55	19.10	10.06	0.76	3.11	0.235	45	24.19	12.16	24.46	12.00	0.27	2.31	0.738	0.50	2.68	0.79	0.650
key pinch D	41	4.24	2.10	4.64	2.10	0.40	0.94	0.027	45	5.28	1.83	5.40	1.90	0.12	0.71	0.738	0.28	0.83	1.57	0.241
key pinch ND	35	4.34	2.53	4.43	2.28	0.09	0.97	0.700	45	4.79	2.01	4.94	2.02	0.14	0.73	0.738	−0.05	0.84	−0.27	0.869
tripod pinch D	40	2.88	1.56	3.00	1.33	0.13	1.18	0.676	45	3.16	1.31	3.26	1.35	0.10	0.82	0.738	0.03	1.00	0.12	0.902
tripod pinch ND	33	2.77	1.79	2.74	1.51	−0.03	0.93	0.877	45	3.00	1.35	2.92	1.46	−0.08	0.83	0.761	0.05	0.87	0.26	0.869
tip-tip pinch D	40	3.25	1.64	3.30	1.57	0.05	0.76	0.768	45	4.22	1.45	4.31	1.62	0.10	0.73	0.738	−0.05	0.74	−0.31	0.869
tip-tip pinch ND	33	3.16	1.92	3.55	1.94	0.39	0.67	0.015	45	3.86	1.64	3.88	1.69	0.03	0.83	0.927	0.36	0.78	2.14	0.107
ARAT D	45	45.71	19.25	49.07	17.90	3.36	7.37	0.015	45	57	0	57	0	0	0		3.36	5.45	3.05	0.023
ARAT ND	41	41.66	22.22	44.83	19.76	3.17	6.39	0.015	45	57	0	57	0	0	0		3.17	4.66	3.18	0.023
NIHSS	43	7.51	5.71	4.74	4.31	−2.77	2.02	< 0.001												
Modified Rankin scale	45	3.69	0.76	3.07	0.99	−0.62	0.65	< 0.001												
FIM	44	75.59	24.58	92.43	23.56	16.84	10.41	< 0.001												

SD, standard deviation; adj, adjusted; D, dominant; ND, non-dominant; NHPT, Nine Hole Peg Test; ARAT, Action Research Arm Test.

As expected, the improvement is more evident when only impaired limbs are considered, as we can see the pre-post differences are often larger in absolute value (Table 5). However, some differences are not significant due to the small sample size, muscle strength improved significantly in the key pinch ND (0.59 ± 0.66, *p*_adj_ = 0.034), and ARAT ND (5.48 ± 8.11, *p*_adj_ = 0.034) of impaired non dominant hand, see Table 5. The improvement is less evident in non-impaired limbs (Table 6).

**Table 5:** Pre-test post-test differences in individuals with stroke on the impaired upper extremity.

	Post-stroke patients with impaired upper extremity							
		PRE		POST		PRE-POST		
	N	Mean	SD	Mean	SD	Mean	SD	*p-*value_adj_
NHPT D	10	57.96	27.93	46.06	21.76	−11.90	21.75	0.157
NHPT ND	10	60.39	31.66	40.27	12.47	−20.11	28.12	0.086
grip D	12	14.96	9.82	14.38	10.95	−0.58	3.16	0.537
grip ND	15	12.07	6.77	13.64	7.45	1.58	3.02	0.094
key pinch D	11	3.36	2.62	4.02	2.78	0.65	0.84	0.068
key pinch ND	15	2.87	1.76	3.46	1.90	0.59	0.66	0.034
tripod pinch D	11	2.12	1.95	2.35	1.60	0.23	1.08	0.537
tripod pinch ND	13	1.72	1.44	2.14	1.55	0.42	0.69	0.086
tip-tip pinch D	11	2.53	2.01	2.73	2.08	0.20	0.67	0.423
tip-tip pinch ND	13	2.01	1.52	2.63	1.75	0.62	0.81	0.053
ARAT D	15	27.33	23.60	34.87	25.68	7.53	10.78	0.053
ARAT ND	21	28.76	24.40	34.24	22.89	5.48	8.11	0.034

SD, standard deviation; adj, adjusted; D, dominant; ND, non-dominant; NHPT, Nine Hole Peg Test; ARAT, Action Research Arm Test.

**Table 6:** Pre-test post-test differences in individuals with stroke on the unimpaired upper extremity.

	Post-stroke patients with unimpaired upper extremity							
		PRE		POST		PRE-POST		
	N	Mean	SD	Mean	SD	Mean	SD	*p-*value_adj_
NHPT D	27	43.41	39.74	33.44	7.41	−9.98	38.67	0.287
NHPT ND	19	32.28	9.44	30.00	7.78	−2.28	4.36	0.212
grip D	28	23.25	8.53	24.79	8.23	1.54	2.68	0.064
grip ND	20	23.03	10.54	23.18	9.95	0.15	3.11	0.907
key pinch D	29	4.62	1.83	4.92	1.80	0.30	0.98	0.269
key pinch ND	20	5.45	2.49	5.17	2.30	−0.28	1.01	0.297
tripod pinch D	28	3.21	1.32	3.29	1.15	0.08	1.26	0.874
tripod pinch ND	20	3.45	1.68	3.13	1.38	−0.32	0.96	0.269
tip-tip pinch D	28	3.58	1.42	3.57	1.31	−0.02	0.80	0.907
tip-tip pinch ND	20	3.90	1.81	4.14	1.86	0.24	0.54	0.234
ARAT D	29	54.93	5.72	56.14	3.39	1.21	3.62	0.250
ARAT ND	20	55.20	6.01	55.95	4.02	0.75	2.17	0.269

SD, standard deviation; adj, adjusted; D, dominant; ND, non-dominant; NHPT, Nine Hole Peg Test; ARAT, Action Research Arm Test.

As expected, clinical characteristics did not change in the control group between the first and second examinations. The pre-post differences between the two groups were significant only in ARAT D and ND, even after adjusting (*p*_adj_ < 0.05) (Table 4).

## Discussion

In this article, we present the results of a sub-study focused on upper limb function. This sub-study is part of the randomized trial NCT05323916, for which the estimated sample size was calculated to be 280 participants. This sample size allows for the demonstration of differences between four groups (70 participants per group) using the WHO Disability Assessment Schedule 2.0 (47). For this sub-study, the sample size was not specifically pre-calculated. However, our sample size matches (4, 23, 48, 49), or even exceeds (1, 15, 50), the sample sizes of other studies addressing this issue.

Although we attempted to assign participants to both groups evenly, perfect 1:1 matching was not possible due to discontinuations from the study for various reasons. This resulted in slightly unbalanced groups: 46 post-stroke patients and 52 controls. Consequently, the groups were analyzed as independent samples in the statistical analysis rather than as matched pairs. To ensure comparability, statistical tests were performed to evaluate whether the groups differed in basic characteristics. Specifically, t-tests were used for age, weight, and height, and chi-square tests were used for sex and laterality.

It is debatable whether we can consider the population selected for our control group to be healthy. Although individuals with neurological or orthopaedic conditions were excluded, the participants were hospitalized, albeit for preventive reasons. They were people of a similar age to those in the experimental group, which suggests that various health issues may be present in such a population.

The age range in our study was quite broad—35 to 89 years for patients and 33 to 93 years for controls. However, most participants fell into the older age categories. Due to the uneven distribution across different age groups, we did not analyze the differences in response to stroke and procedures performed in our study with respect to age. As well, due to low samples, we did not analyze the differences with respect to gender.

Our hypothesis that individuals with stroke have greater upper extremity impairment than controls was confirmed. However, we were surprised to find that significant differences in muscle strength between stroke patients and controls were evident only after adjusting for grip, key, and tip-tip pinch strength in the dominant hand (regardless of impairment). These findings did not show more pronounced differences compared to the control group, as we had expected to see greater disparities across a broader range of parameters.

Only few studies (49, 51) consider the dominance of the limbs on the affected side in individuals with stroke, although it may play a role. Most studies compare impaired and unimpaired upper extremities independently on upper extremity dominance (10, 36, 43, 49). Gilbertson and Barber-Lomax (52) found no significant difference between the dominant and non-dominant upper limbs; however, the variability may be greater in the values for the non-dominant upper limb, making it more difficult to detect significant differences with medium-sized samples.

Across studies, stroke survivors have lower muscle strength, but this varies depending on neurological deficit, post-stroke time, and other factors. For example, Lang et al., (49) assessed individuals in the acute phase (±9.5 compared to our study ±21.6 days post-stroke) with a lower neurological deficit on NIHSS (5.3 ± 1.8 compared to our study 7.51 ± 5.71) and documented lower grip strength (9.6 ± 10.5 compared to our study 20.55 ± 9.51). Similar values of the affected grip and pinch as in our study documented by Chen et al., (43) in people with similar upper limb impairment according to NHPT (60.1 ± 38.2 compared to our study 57.96 ± 27.93).

The values between studies are essentially incomparable because authors use different units (36, 40, 53), different methodologies (10), use normalized relative strength (10). Although it is recommended not to use the unaffected upper extremity as a control group (8), only a few studies compared results with a healthy control group. For comparison, it is also possible to use normative data (52, 53); for example, compared to British norms, our controls have lower muscle strength (52). However, our controls cannot be considered a healthy population because they were hospitalized for some reason in our hospital, although serious neurological and orthopedic diseases were excluded.

While the difference when comparing muscle strength parameters between individuals with stroke and controls in our study is not significant for non-dominant hand, clinical tests (ARAT, NHPT) show significant differences for both dominant and non-dominant hands.

COMIRESTROKE was associated with an improvement of both primary and secondary outcomes. The primary outcomes improved significantly, even after adjustment: tip-tip pinch of the impaired non-dominant hand, and ARAT of both dominant and non-dominant hand.

Demonstrating correlations between primary and secondary outcomes further confirms the consistency of our findings and their alignment with existing literature (54, 55). The relationship between the ARAT and the FIM has been explored in detail by Rabadi and Rabadi (55). The authors appropriately addressed multiple comparisons, considering the established collinearity between the NHPT and the ARAT (54), as well as the well-documented relationships between grip strength, the ARAT, and the NHPT.

When comparing our results to published Minimal Clinically Important Differences (MCID), they are less compelling, highlighting another limitation of this study. Specifically, for grip muscle strength, there was no significant change, with our findings being well below the MCID: 5 kg for the dominant hand (our study showed a change of −0.58 kg) and 6.2 kg for the non-dominant upper extremity (our study showed a change of 1.58 kg) (49). Unfortunately, we cannot evaluate changes in pinch strength in the context of MCID, as these values have not yet been established.

The greater improvement of the affected limb, which was non-dominant, surprised us and contradicted with opinion of Langan and van Donkelaar (50), who thought that people with the dominant hand affected by stroke have an advantage in improving the mobility of the more affected hand compared to those individuals with the non-dominant hand affected by stroke. Our results, on the other hand, align with a study where the highest median percent improvement in the affected non-dominant upper extremity was observed for grip strength (51).

We attribute this to the fact that in our study, we did not provide targeted therapy for upper extremity function but rather a comprehensive intensive rehabilitation. As a result, the rehabilitation did not specifically focus on improving upper extremity function or on the unique roles of each limb, such as enhancing dexterity in the dominant upper extremity or strengthening the non-dominant upper extremity for stabilization.

However, we consider the improvement in primary outcomes, mainly in ARAT (from 27.33 ± 23.60 to 34.87 ± 25.68), to be important, especially because it occurred after a complex intensive program that was not aimed at improving upper extremity function. The mean improvement of ARAT in our study corresponds to the improvement in the experimental group in other studies (15, 23) evaluating the effect of the targeted treatment on upper extremity function (the Repetitive Facilitative Exercise Program). On the other side, another kind of targeted therapy, aimed at improving the function of the affected upper extremity (low and high intensity of Constraint-induced movement therapy) can lead to significant improvement of ARAT (from 19.65 ± 3.73 to 36.20 ± 4.05 in 14 days) (48). Notably in an upper limb targeted therapy patients were stratified by the severity of the post-stroke arm-hand impairment and found that the sub-group of patients with a moderately affected arm-hand presented with best results regarding the ARAT and the Fugl-Meyer Motor Assessment (56).

Our results are consistent with the literature and confirm that ARAT is a suitable tool to predict improvement in individuals with stroke (9, 57), despite the fact that this assessment is limited by the use of an ordinal scale (3). However, its advantages are strong psychometric properties, fast administration (7) and bringing information about a patient’s upper limb capacity by mimicking activities of daily living (57).

The greatest improvement was observed in the secondary outcomes: neurological impairment measured by the NIHSS, disability assessed using the MRS, and functional independence evaluated by the FIM. The improvement in FIM in our study (16.84 ± 10.41) was greater than in a study that reported a mean difference of 2.70 (4), or in a study where the change was 1.00 (1). Both studies (1, 4) specifically aimed to influence upper limb function while simultaneously assessing the impact on independence using FIM. However, the MCID, defined as 22 for stroke survivors (58), was not achieved in our study either.

Although the probands in both studies (1, 4) were young (50.7–55.51 years versus 70.25 ± 12.66 in our study), probably due to the severity of the disability (114.3–119.4 points on FIM versus 75.59 ± 24.58 point in our study), the potential for improvement has not been exploited. Although they were offered targeted therapy for upper limb function (robot-assisted therapy (1), Constraint-Induced Therapy Versus Bilateral Arm Training (4)) that resulted in improvement, we are unable to compare the outcomes as the authors used different clinical tests than we did. Their therapy (1, 4) did not affect self-sufficiency and probably not the degree of neurologic disability (which, unfortunately, we cannot compare).

In our opinion, the timeliness of therapy plays a major role in the possibility of recovery. This is possibly due to the appropriate timing of complex intensive rehabilitation into the sub-acute phase (16–18, 30) when spontaneous processes (3, 19–21) leading to recovery can be suitably enhanced (6, 22).

Since in our study we do not evaluate the effect of any targeted therapy on the function of the upper limbs, we can attribute their improvement to the complexity and intensity of the rehabilitation.

Among the strengths of our study is the use of validated outcome measures, a high completion rate among participants, and a relatively high number of participants—our sample matches (4, 23, 48, 49) or even exceeds (1, 15, 50) the studies dedicated to this issue.

Among its weaknesses is missing FIM, measuring self-efficiency, in the control group. As secondary outcomes, parameters related to neurological deficits were chosen. However, the analyses showed that the FIM score in the control group would be useful for comparing the difference in therapy effect between groups.

Despite the limitations of the current study, we believe it provides valuable resource for future research. In this study, we described impaired muscle strength and dexterity in individuals with stroke in comparison to controls. We showed that muscle strength and dexterity may affect their independence. Furthermore, we found, a very important finding for clinical work, that impaired muscle strength and dexterity can be improved by a comprehensive intensive rehabilitation program (not only by means of specific therapy aimed at improving the function of the upper limbs, as is often documented in the literature). This program was even associated with lowered neurological disability and improved self-sufficiency. Further studies are needed to study effect of individual physiotherapeutic techniques combined in COMIRESTROKE and to compare its effect with other treatments.

## Data Availability

The raw data supporting the conclusions of this article will be made available by the corresponding author upon request.
